# Targeting histone methyltransferases and demethylases in clinical trials for cancer therapy

**DOI:** 10.1186/s13148-016-0223-4

**Published:** 2016-05-24

**Authors:** Ludovica Morera, Michael Lübbert, Manfred Jung

**Affiliations:** Institute of Pharmaceutical Sciences, Albert-Ludwigs-University Freiburg, Albertstraße 25, 79104 Freiburg, Germany; Department of Hematology and Oncology, University of Freiburg Medical Center, Hugstetter Straße 55, 79106 Freiburg, Germany; German Cancer Consortium (DKTK), Freiburg, Germany

**Keywords:** Methylome, Lysine methylation, Histone methyltransferase, Histone demethylase, Clinical trial, Methyltransferase inhibitors, Demethylase inhibitors, Epigenetics, Histone modifications

## Abstract

The term epigenetics is defined as heritable changes in gene expression that are not due to alterations of the DNA sequence. In the last years, it has become more and more evident that dysregulated epigenetic regulatory processes have a central role in cancer onset and progression. In contrast to DNA mutations, epigenetic modifications are reversible and, hence, suitable for pharmacological interventions. Reversible histone methylation is an important process within epigenetic regulation, and the investigation of its role in cancer has led to the identification of lysine methyltransferases and demethylases as promising targets for new anticancer drugs. In this review, we describe those enzymes and their inhibitors that have already reached the first stages of clinical trials in cancer therapy, namely the histone methyltransferases DOT1L and EZH2 as well as the demethylase LSD1.

## Background

All cells within one individual contain the same genetic information in the DNA; however, gene expression and hence phenotypes vary widely in different cells and tissues. In the nucleus, the DNA is packaged together with structural proteins (histones) to form a complex known as chromatin. Chromatin can appear in a condensed, transcriptionally repressed form (heterochromatin) or in a generally decondensed, and transcriptionally active form (euchromatin). The local regulation of chromatin state is believed to control accessibility to DNA, allowing, respectively, control of transcription, replication, recombination, and DNA repair. Different epigenetic mechanisms affect the chromatin state. These consist of histone post-translational modifications (PTMs) [1, 2], DNA modifications [3], replacement of canonical histones with histone variants [4], ATP-dependent nucleosome remodeling [5, 6], non-coding RNA (ncRNAs) [7], and others [8–10]. Here, we will focus on histone modifications, specifically reversible histone methylation.

**Fig. 1:**
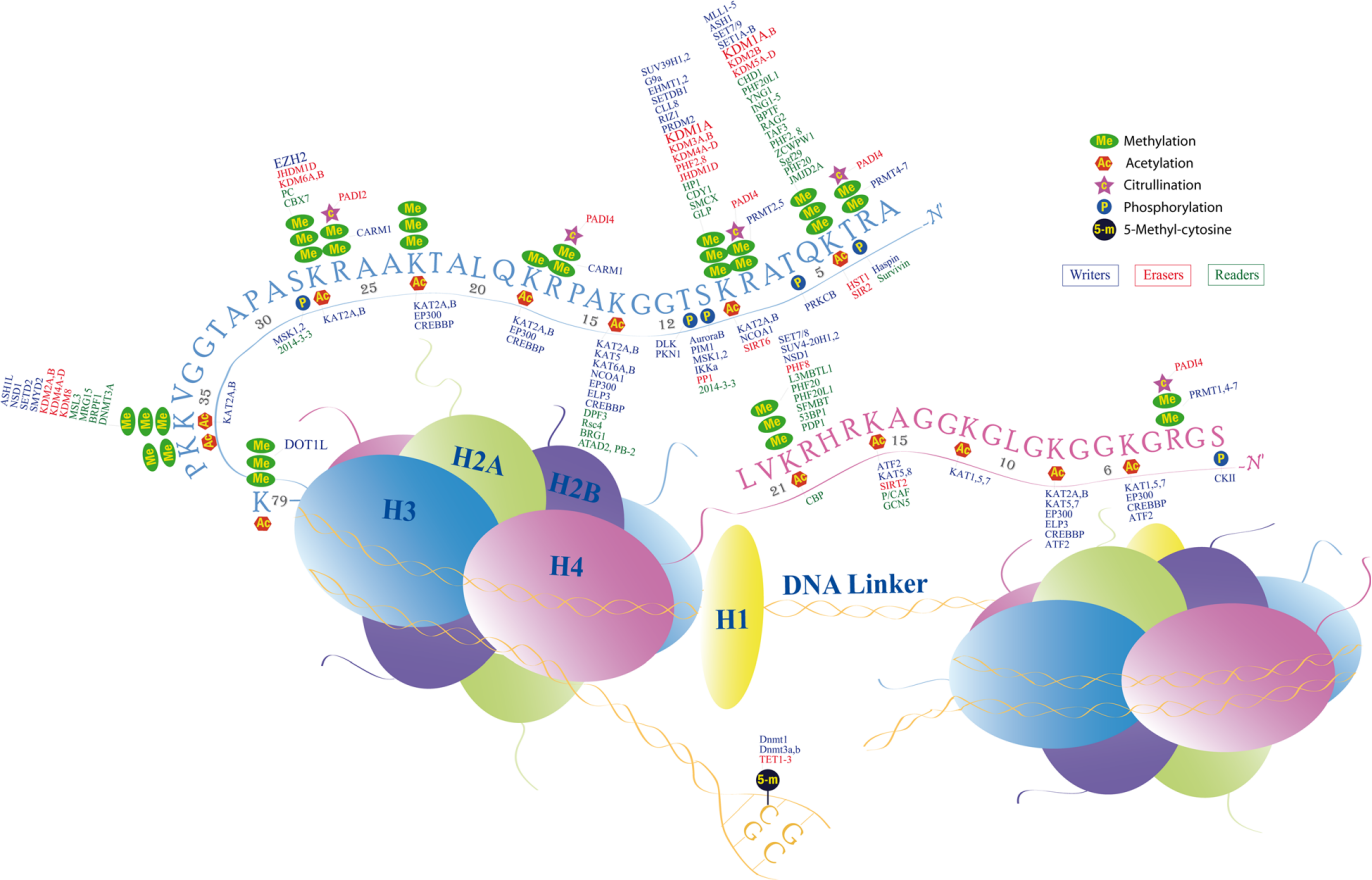
Nucleosome structure and principal modification sites on H3, H4, and DNA. The reported writers, erasers, and readers for these modifications are also depicted

A nucleosome, the repeating unit of chromatin, is composed of a histone octamer core, which consists of two copies of each histone H2A, H2B, H3, and H4 proteins, and a short segment of DNA, between 145 and 147 base pairs, which is wrapped around it (Fig. 1). The repeating nucleosome cores further assemble into higher order structures which are stabilized by the linker histone H1 [11]. The core is predominantly globular except for the histone tails (~30 amino acids) protruding from them. A wide range of PTMs occurs not only at the histone *N*-terminal tails, including acetylation, methylation, phosphorylation, ubiquitination, SUMOylation, crotonylation, and others [12] but also in the core of the histones and in the C-terminal regions [13, 14]. The enzymes responsible of the addition of chemical groups onto either histone tails or the DNA itself are commonly termed “writers”, the proteins that recognize these specific epigenetic marks are called “readers”, and then, since the epigenetic modifications are not permanent, the “erasers” can remove them. In histone tails, lysine and arginine residues are the main sites of modifications (principally acetylation and methylation). It is interesting to note that several histone lysines can be substrates of methylation as well as of acetylation processes (Fig. 1). A balance between these two competitive modifications at H3K9 is, for example, required for chromosome segregation [15]. While the acetylation of the lysine directly abolishes the positive charge of the amino acid, eliminating the electrostatic bond between histones and DNA, thus allowing the euchromatin formation, histone lysine and arginine methylation do not alter the charge [16]. These modifications influence instead the binding of chromatin-associated proteins; different readers that specifically recognize these modifications have been described [17].

The enzymatic methylation of histones is performed by lysine methyltransferases (KMTs) and arginine methyltransferases (PRMTs), with *S*-adenosyl-l-methionine (SAM) as the methyl donor. Histone methylation can involve the transfer of up to three methyl groups, thus resulting in mono-, di-, or trimethylated lysine, respectively, and in mono- or di- (asymmetric or symmetric) methylated arginine. Surprisingly, the same modifications could also lead to opposite activities (e.g., H3K4me2 and H3K4me3) probably due to the recruitment of different effector proteins by the readers [18, 19]. Hence, the discussion around the existence of a histone code [20] has lately shifted to calling it rather a language which emphasizes the context dependence of the modifications [2].

The demethylation of lysines was for a long time thought to be irreversible, until Shi et al. reported in 2004 that the amine oxidase lysine-specific demethylase 1A (LSD1; also known as KDM1A) was able to specifically demethylate histone H3 lysine 4 (H3K4) [21]. For arginines, the existence of a “true” demethylase remains to be proven [22]. Arginine as well as mono-methylated arginine can, however, be converted to citrulline by the protein-arginine deiminases (called PADs or PADIs) [23].

The most extensively studied histone lysine methylation sites are H3K4, H3K9, H3K27, H3K36, H3K79, and H4K20 (Table 1), although many methylated-lysine residues have been found also in H1, H2A, H2B, and in further positions within H3 and H4. While some lysine methylation marks are preferentially associated with euchromatin and hence gene activation (like H3K4, H3K36, and H3K79) or with heterochromatin and gene silencing (H3K9, H3K27, and H4K20) [24], more often the final effect on chromatin is influenced by the interplay of several histone modifications together (“histone crosstalk”) [25].

**Table 1:** Principal writers and erasers of methyl lysines in histone 3 and 4 and their association with cancer

	H3K4	H3K9	H3K27	H3K36	H3K79	H4K20
Writers	MLL1 (KMT2A) MLL2 (KMT2B) MLL3 (KMT2C) MLL4 (KMT2D) MLL5 (KMT2E) SET1A (KMT2F) SET1B (KMT2G) ASH1 (KMT2H) SMYD1 (KMT3D) SMYD2 (KMT3C) SMYD3 (KMT3E) SET7/9 (KMT7)	SUV39H1 (KMT1A) SUV39H2 (KMT1B) G9a (KMT1C) GLP (KMT1D) SETDB1 (KMT1E) SETDB2 (KMT1F) RIZ1 (KMT8)	EZH2 (KMT6)	SET2 (KMT3A) SET3 NSD1 (KMT3B) NSD2 NSD3 SMYD2 (KMT3C) SETMAR	DOT1L (KMT4)	SET8 (KMT5A) SUV420H1(KMT5B) SUV420H2 (KMT5C) NSD1(KMT3B)
Erasers	LSD1(KDM1A) LSD2 (KDM1B) JHDM1A (KDM2A) JHDM1B (KDM2B) JARID1A (KDM5A) JARID1B (KDM5B) JARID1C (KDM5C) JARID1D (KDM5D) NO66	LSD1 (KDM1A) JHDM2A (KDM3A) JHDM2B (KDM3B) JHDM3A (KDM4A) JMJD2B (KDM4B) JMJD2C (KDM4C) JMJD2D (KDM4D) JHDM1D (KDM7) PHF8	UTX (KDM6A) JMJD3 (KDM6B)	JHDM1A (KDM2A) JHDM1B (KDM2B) JMJD2A (KDM4A) JMJD2B (KDM4B) JMJD2C (KDM4C)	Not described	Not described
Cancer association	MLL rearrangement in leukemias and in different solid tumors [172, 173]. SMYD3 overexpressed in colorectal and hepatocellular cancers [174]. JARID1B overexpressed in breast cancer [175]. LSD1 overexpressed in prostate cancer [176].	G9a involved in suppressor gene silencing [177]. SUV39H1, increased mRNA in colon cancer [178]. SETDB1 amplified and overexpressed in melanoma [179]. Downregulation of LSD1 impairs androgen-dependent proliferation of prostate cancer cells [140].	EZH2 amplified in multiple solid tumors [84, 180] and mutated in lymphomas [110] and myelodysplastic syndromes and myeloproliferative neoplasms [181]. H3K27 mutated in glioblastoma[182].	Translocation and overexpression of NSD3 in AML and breast cancer [183, 184] and of NSD1 in prostate cancer [185] and in neuroblastoma [186].	*MLL*-rearranged leukemias	Loss of H4K20me3 in non-small cell lung cancer [187] and in several cancer cell lines [27, 188].

An aberrant covalent histone modification profile, leading to a dysregulated expression of oncogenes and tumor suppressor genes, is often associated with cancer [26]. Fraga et al. demonstrated, for example, that the reduction of Lys16 acetylation and Lys20 trimethylation at histone 4 constitutes a typical “cancer signature” [27]. Furthermore, aberrant histone methylation has been related not only with cancer but also with mental retardation and aging [28–30].

DNA methylation and histone acetylation were among the first epigenetic targets to be addressed for drug development and several inhibitors of DNA methyltransferases, and histone deacetylases are approved by the Food and Drug Administration (FDA) for clinical use in cancers [31]. In contrast, histone methylation still offers a large room for discovery and pharmacological interventions, but lately, the first inhibitors have also reached clinical testing. This review focuses on the recent reports on clinical trials of compounds targeting reversible histone lysine methylation and the biology behind their targets. Some of this information is not yet published in peer-reviewed journals, so statements on the clinical activity of these inhibitors have to be viewed with caution in these cases.

## Review

### Lysine methyltransferases

Up to date more than 50 lysine human methyltransferases (KMTs) have been reported. These enzymes possess high selectivity concerning the histone lysine residue they target, as well as the degree of methylation they can confer. There are two different families of lysine methyltransferases divided on the basis of their catalytic domain sequence: the DOT1-like proteins and the SET domain-containing proteins. The acronym SET came from the *Drosophila* polycomb proteins in which this domain was originally found, namely suppressor of variegation 3–9 (Su(var)3–9), enhancer of zeste (E(z)), and trithorax (Trx) [32–34]. These methyltransferases methylate lysines in histones as well as in non-histone substrates [35]. The KMT SET7/9, for example, can stabilize the tumor suppressor p53 by methylation at K372 [36]. It methylates also other non-histone substrates, like the DNA methyltransferase 1 (DNMT1), estrogen receptor alpha (ERα), and nuclear factor NFκB [37]. Among the KMTs, the human DOT1-like (DOT1L) protein is the only one which does not possess a SET domain, and its catalytic domain is structurally more similar to the arginine methyltransferases [38, 39].

Based on the sequence similarity in their SET domain and in adjacent protein regions, the SET demethylases can be divided into four families: SET1, SET2, SUV39, and RIZ [40, 41]. These methyltransferases generally function in multiprotein complexes. The SET methyltransferase represents the catalytic domain, while the accessory proteins control the selectivity and the activity of the complex. The SET1 family is characterized by the presence of the SET domain usually followed by a post-SET domain, even if the two most studied members of this family, EZH1 and EZH2, do not harbor this region. The members of the SET2 class have a SET domain that is always between a post-SET and an AWS domain, rich in cysteines. In this family, we find the nuclear receptor binding SET domain-containing proteins NSD1-3, the SETD2 and the SMYD family proteins. The SUV39 family members all present a pre-SET domain, essential for enzymatic activity [32]. SUV39H1, SUV39H2, G9a, GLP, ESET, and CLLL8 belong to this class. Finally, the RIZ family members, bearing the SET domain at the amino terminus, are RIZ1, BLIMP1, and PFM1.

In addition to these families, there are other SET domain-containing methyltransferases which have not been assigned to a certain group, like SET7/9, SET8, SUV4-20H1, and SUV4-20H2 [41]. Here, we highlight those lysine methyltransferases for which the first inhibitors are in clinical trials, more extended reviews can be found elsewhere [26, 42, 43].

## DOT1L

DOT1L protein is the mammalian homologue of disruptor of telomeric silencing-1 (Dot1), a gene found in *Saccharomyces cerevisiae* [44]. DOT1L is the only enzyme responsible for mono-, di-, and trimethylation of the *ε*-amino group on H3K79, an activating mark with respect to gene transcription [33, 45]. The turnover of this modification is generally slow and no KDM able to remove this mark has been reported so far [46]. It has been suggested that the monoubiquitinylation of H2BK120 stimulates the H3K79 methyltransferase activity of DOT1L [47, 48]. Min et al. were able to solve the structure of the catalytic domain of human DOT1L in complex with the methyl donor SAM at 2.5 Å, and a few years later, a 2.1 Å crystal structure was reported [38, 49].

DOT1L plays a crucial role in various physiological and pathological processes, like transcriptional regulation, cell-cycle regulation, DNA repair, embryonic development, hematopoiesis, cardiac function, and leukemia development [39, 50–55]. Even if, to date, no genomic alterations of DOT1L have been directly implicated in cancer, this methyltransferase is a promising pharmacological target for the treatment of a unique group of leukemias, which presents a chromosomal translocation of the mixed-lineage leukemia (MLL) gene (chromosome 11q23). Examples are the acute myeloid leukemias (AML), the acute lymphoblastic leukemias (ALL), and the biphenotypic (mixed lineage) leukemias (MLL). These aggressive leukemia forms constitute more than 70 % of infant leukemias and about 10 % of adults’ leukemias and are associated with poor prognosis for the patients: children affected by ALL harboring this translocation have an overall survival of 50 %, whereas children with ALL that does not harbor the MLL translocation have an overall survival of over 80 % [56–59]. The *MLL* gene normally encodes for a SET domain KMT (MLL1) which performs the methylation of H3K4 [60]. When MLL is translocated, the catalytic methyltransferase SET domain is lost and the remaining MLL protein is fused with a variety of partners known as MLL translocation fusion proteins (like AF4, AF9, AF10, and ENL) [61–63]. These fusion partners are able to recruit DOT1L. Also, the nature of the fusion proteins can influence the prognosis of the MLL-rearranged leukemias; in particular, the association of MLL with AF10 is associated with very poor outcomes [64]. These new translocation product proteins retain, thus the gene recognition elements of MLL, with the added ability to recruit DOT1L. The resulting increased H3K79 methylation is a positive transcription mark that, bypassing the normal transcription regulation, causes the expression of proleukemogenic genes (like *HOXA9* and *MEIS1*), and thus the development of leukemia [65–67]. A unique H3K79 methylation profile characterizes the MLL-rearranged leukemias in comparison to the germline MLL leukemias [61]. In several in vitro studies, MLL-fusion-transformed cells, in which the expression of DOT1L was suppressed or inactivated, showed differentiation and apoptosis [68, 69]. These studies then support the hypothesis that the inhibition of DOT1L could be a promising therapeutic strategy for the treatment of MLL-rearranged leukemias.

**Fig. 2:**
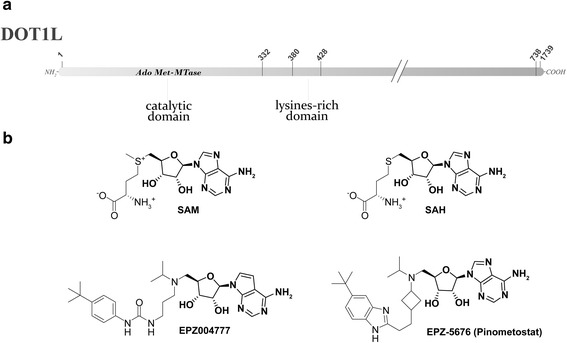
**a** Schematic view of DOT1L principal domains. **b** Structures of the methyl donor SAM, its enzymatic product SAH and of two DOT1L inhibitors. The SAM-like shared moiety is *highlighted in bold*

Small molecules targeting DOT1L were designed using the cofactor SAM or the enzymatic product *S*-adenosyl-l-homocysteine (SAH) as the starting point (Fig. 2). Generally, there are four classes of inhibitors: the SAH-like, the mechanism-based, the carbamate-containing, and the urea/benzimidazole-containing compounds. All of them share a common adenosine or deazaadenosine group, in analogy with the enzyme cofactor SAM [70–77]. In 2011, Epizyme Inc. reported EPZ004777 as the first potent and selective inhibitor (Fig. 2) [72]. Crystal structures of this inhibitor and some analogs within DOT1L were reported [73, 75]. EPZ004777 shows a remarkable selectivity against other histone methyltransferases, which also use SAM as cofactor. EPZ004777 was able to selective kill MLL-rearranged leukemia cells in culture, while having little effect on non-MLL translocated cells, and prolong survival in mouse model of MLL-rearranged leukemia [72, 78]. However, despite these results, its poor pharmacokinetic properties made this compound unsuitable for clinical development. In a second generation of inhibitors, a novel derivative of EPZ004777 was reported, in which the ribose moiety was replaced with a cyclobutyl ring (EPZ-5676, Fig. 2), to improve pharmacokinetic properties [77]. EPZ-5676 shows the same binding mode as its parental compound, with an improved activity against DOT1L (EPZ-5676 Ki <0.08 nM; EPZ004777 Ki = 0.3 nM), a much-extended drug-target residence time and a 37,000-fold selectivity against other protein methyltransferases [77]. Both inhibitors showed a good activity against the proliferation of some leukemia cell lines with *MLL* translocation, as MV4-11 (*MLL-AF4*), MOLM-13 (*MLL-AF9*), and THP1 (*MLL-AF9*), with little effect on leukemia cells lacking this translocation [71, 72, 77]. Despite the pharmacokinetic improvements, EPZ-5676 still showed a low oral bioavailability [79]. Continuous infusion of EPZ-5676 (70 mg/kg per day) for 21 days achieved complete and sustained tumor regressions (more than 30 days after the end of treatment period) in a nude rat subcutaneous xenograft model of MLL-rearranged leukemia. Interestingly, these doses were also well tolerated with no overt signs of toxicity in experimental animals. Reducing the length of treatment to 14 days or the dose to 35 mg/kg per day still caused sustained tumor regression, but with less efficacy [77]. EPZ-5676 was also found to act synergistically with cytarabine, daunorubicin, and the DNMT inhibitor azacitidine, three common AML standard care drugs, in the human acute leukemia cell lines MOLM-13 (*MLL-AF9*) and MV4-11 (*MLL-AF4*) [80].

A first-in-human study of EPZ-5676 (now termed Pinometostat) is currently in a phase I trial in adults with AML and ALL with rearrangements of the MLL gene (ClinicalTrials.gov identifier: NCT01684150). A first part of the study with dose escalation, to determine the maximum tolerated dose (MTD) and the recommended phase 2 dose (RP2D), has been completed. Currently, this study is in the expansion phase, and patients are receiving, in cycles of 28-day, continuous IV infusion of EPZ-5676. The study has been completed in November of 2015. A phase I trial of EPZ-5676 recently opened for pediatric patients with relapsed/refractory leukemias bearing a rearrangement of the *MLL* gene (ClinicalTrials.gov identifier: NCT02141828). The expected completion for primary outcome is May 2016.

## EZH2

Enhancer of zeste homologue 2 (EZH2) belongs to the SET1 family of methyltransferases. It is the catalytic component of the polycomb repressive complex 2 (PRC2). Polycomb repressor complex 1 and 2 (PRC1 and PRC2, respectively) are transcriptional repressors [81, 82]. They are involved in cellular memory, X-chromosome inactivation, cancer metastasis, cell proliferation, and cell differentiation via epigenetic histone modifications [83, 84]. Gene silencing is achieved for PRC1 via ubiquitylation of H2AK119, while PRC2 exhibits histone lysine methyltransferase activity through its catalytic subunit, represented by EZH2 or its close homologue EZH1 [83, 85–87]. PRC2 performs three successive methyl transfer reactions, producing ultimately H3K27me3. EZH1 and EZH2 are the only enzymes known to catalyze this epigenetic transformation. The repressive effects of the polycomb complexes are counteracted by the trithorax group proteins, a group of transcriptional activators [88]. PRC2 consists of several subunits, among them there are EZH2, embryonic ectoderm development (EED), and the suppressor of zeste 12 (SUZ12) [89]. Interestingly, EZH1/EZH2 lack enzymatic activity as isolated proteins, in fact, they are able to methylate lysine residues only when they are in complex with EED and SUZ12 [83, 90]. In addition to these three subunits, PRC2 can bind other subunits, like AEBP2, which regulates the activity or the localization of the complex.

PRC2 seems to be required for the activity of PRC1 on H2AK119. The exact mechanism is not completely understood, but likely PRC2 performs H3K27 trimethylation on target genes for the initiation of silencing. Then PRC1 is recruited to these genes to consolidate the silent state through ubiquitylation [91, 92]. PRC2 is also able to interact, through the EED subunit, with other histone modifiers, like histone deacetylases (HDAC) and DNA methyltransferases (DNMT). Since H3K27 could also bear an acetyl group, an initial HDAC activity is required before EZH2-mediated H3K27 methylation. The PRC2 can then also associate with different DNMTs, which perform cytosine methylation, then resulting in gene silencing [93]. In summary, a model of polycomb gene silencing, initiated by PRC2, and maintained by PRC1, could be represented by histone deacetylation, followed by histone methylation and DNA methylation [91].

**Fig. 3:**
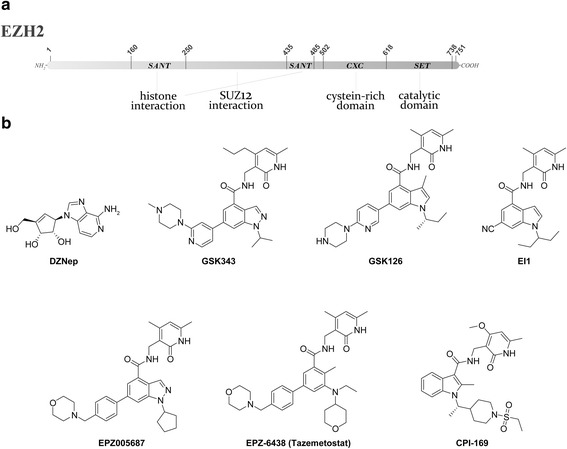
**a** Schematic view of EZH2 principal domains. **b** Structures of EZH2 inhibitors

Several studies show that EZH2 deregulation is frequently associated to poor prognosis in solid tumors, including the prostate, breast, kidney, and lung [94–98]. EZH2 overexpression is also associated with metastasis, tumor progression, and poor clinical outcome [99, 100]. Different mechanisms were reported as cause of increased EZH2-dependent signaling in tumor cells, like gene mutations [101], amplification [102], certain transcriptional signals and pathways [103–105], hypoxia [106], and multiple microRNAs [107–109]. Heterozygous Tyr 641 mutations in the catalytic EZH2 SET domain, for example, were also identified in some myeloid malignancies, especially in follicular lymphoma (7.2 %) and in diffuse large B cell lymphoma (DLBCL) (21.7 %) that derive from germinal center B cells [110]. Initially, it was thought that this mutation caused loss of EZH2 methyltransferase activity, but later on was shown to modulate the substrate specificity and to increase H3K27me3 [111]. Regardless of the molecular mechanism involved, EZH2 overexpression leads to higher levels of the repressive H3K27me3 mark, responsible for the silencing of tumor suppressor genes in cancer cells. Several inhibitors of EZH2 have been reported (Fig. 3). One of the most studied compounds is 3-Deazaneplanocin A (DZNep), a derivative of the antibiotic neplanocin-A [112, 113]. DZNep is not a direct EZH2 inhibitor, but rather a SAH-hydrolase inhibitor. The increase of the intracellular SAH concentration leads to the degradation of the PRC2 complex by a feedback inhibition mechanism [114]. DZNep was able to reactivate PRC2 target genes, thus mediating apoptosis in cancer cells, like brain, breast, colorectal, liver, lung, and prostate cancer cells, but not in normal cells [112, 115]. Given the pleiotropic action of this inhibitor, its use as a chemical probe, for specifically studying the EZH2 contribution in the PRC2 overall activity, is very limited. Still, such a multimodal inhibitor could be become a valuable drug [116], but further rational optimization for second generation drugs is difficult in such a case. More recently, research groups at GSK, Novartis, and Epizyme have identified new hits for EZH2 inhibition from high-throughput screening, and optimized them subsequently. Many of them share a pyridone scaffold and the mechanism of action, namely competition with the cofactor SAM. However, since there is not any EZH2-inhibitor co-crystal structure (Wu et al. published a 2.0 Å crystal structure of EZH2, without the cofactor or substrate) [117], this mechanism of action is, for the moment, only supported by the enzymology data. It is interesting to notice that the effects of EZH2 inhibition are time dependent. Given the slow kinetics of H3K27me3 turnover, it is not surprising that only a prolonged EZH2 inhibition (several days) is able to cause a H3K27me3 reduction, sufficient to alter the gene expression [118]. Of note is that the SAM competitive inhibitors are effective against cell lines bearing gain-of-function EZH2-mutations (Tyr641 or Ala677), even if they induce a decrease of H3K27me3 in both EZH2-mutated and wild-type cancer cells [97]. We will focus particularly on the inhibitors currently in clinical trials (Fig. 3). GSK343 demonstrated good activity against EZH2, in both enzymatic and cellular assays (EZH2 Ki app = 1.2 nM, H3K27me3 cell IC_50_ = 174 nM in HCC1806 cells) [119]. It displays a very high selectivity, more than 1000-fold, against other methyltransferases, and of 60-fold against EZH1, which possesses a 96 % sequence identity of the catalytic SET domain with EZH2. More recently, a new inhibitor from GlaxoSmithKline was reported (GSK126), which is the most potent EZH2 inhibitor (Ki app 0.3 nM, 150-fold selectivity against EZH1) reported so far. GSK126 was able to effectively inhibit the proliferation of EZH2-mutant DLBCL cell lines and displayed a robust activity in mice xenograft models of DLBCL bearing EZH2-activating mutations [120]. In April 2014, GlaxoSmithKline began a phase1/2 dose escalation study to investigate the safety, pharmacokinetics, pharmacodynamics, and clinical activity of GSK2816126 (GSK126) in patients with relapsed or refractory diffuse large B cell and transformed follicular lymphoma (ClinicalTrials.gov identifier: NCT02082977). This study will determine the recommended phase 2 dose (RP2D) for GSK2816126 given i.v. Novartis reported EI1 (Fig. 3), an EZH2 inhibitor which also binds to the SAM pocket of EZH2. It is highly potent (EZH2 Ki = 13 nM) and selective (>10,000-fold against other histone methyltransferases and about 90-fold against EZH1) [121]. In 2012, Epizyme reported a potent EZH2 inhibitor (EPZ005687, Fig. 3) with a Ki of 24 nM, and >500-fold selectivity against other methyltransferases and 50-fold against EZH1. EPZ005687 selectively inhibits H3K27 methylation of lymphoma cells harboring heterozygous EZH2 mutations at Tyr641 or Ala677, with minimal effects on proliferation on wild-type cells [122]. One year later, the same group reported EPZ-6438 (tazemetostat, formerly known also as E7438, Fig. 3), with superior potency (EZH2 Ki = 2.5 nM) and good oral bioavailability. EPZ-6438 demonstrated also robust in vivo activity in a EZH2-mutant non-Hodgkin lymphoma (NHL) mice xenograft model, causing dose-dependent tumor growth inhibition. Two EZH2-mutant xenograft models in mice dosed orally with EPZ-6438 for 28 days remained tumor free for up to 63 days after stopping compound treatment [123, 124]. In June 2013, a phase 1/2 clinical trial of tazemetostat has started in patients with advanced solid tumors or with relapsed or refractory B cell lymphomas (ClinicalTrials.gov identifier: NCT01897571). The first part (dose escalation and dose expansion phases) of this phase 1/2 trial is now completed and EPZ-6438 showed a favorable safety and tolerability profile, with the majority of adverse events of grade 1 or grade 2, in particular asthenia, anorexia, anemia, dyspnea, and nausea. Nine of 15 evaluable NHL patients achieved an objective response, with two complete responses and seven partial responses. One patient, evaluated for EZH2 status, possessed a specific EZH2 tumor mutation (histidine instead of tyrosine 646, Y646H). This patient achieved a partial response after 16 weeks of therapy and will remain on study. An 800-mg dose twice per day is confirmed as the recommended phase 2 dose. Pre-clinical data show a synergism between tazemetostat and R-CHOP (rituximab, cyclophosphamide, doxorubicin, vincristine, and prednisone) and between tazemetostat and a not yet disclosed B cell signaling pathway inhibitor in DLBCL (Epizyme, International Conference on Malignant Lymphoma (ICML), Recap Presentation June 22, 2015). In November 2015, Epizyme began a phase 1 study of tazemetostat in pediatric subjects with relapsed or refractory integrase interactor 1 (INI1) negative tumors or synovial sarcoma (ClinicalTrials.gov identifier: NCT 02601937) and a phase II study for adult patients with a similar cancer (ClinicalTrials.gov identifier: NCT 02601950).

Treatment with EPZ-6438 caused also apoptosis in cell lines and dose-dependent tumor regression in xenograft model of malignant rhabdoid tumors with mutated SMARCB1 (also known as SNF5, INI1, and BAF47), a subunit of the SWItch/sucrose non-fermentable (SWI/SNF) chromatin remodeling complex [124]. The SWI/SNF complex consists of approximately 15 subunits and contributes to transcriptional regulation and DNA repair. It has been shown that at least nine of its subunits are frequently mutated in a wide variety of cancers (20 % of all human tumors) [125]. In addition to SMARCB1, other SWI/SNF subunits are often mutated in cancer like ARID1A in ovarian carcinoma [126], SMARCA4 (also known as BRG1) in lung and pancreas cancer [127, 128], and PBRM1 in renal cancer [129]. It has been demonstrated that the inactivation of these subunits renders the cancer cells functionally dependent on the EZH2 catalytic activity, and the treatment with EZH2 inhibitors gave very promising results against tumors harboring SWI/SNF mutations [124, 126]. However, very recently, Kim and co-workers demonstrated that the SWI/SNF mutant cancer cells are only partially dependent on the EZH2 histone methyltransferase activity; they suggest that the dependence on EZH2 could arise from a non-enzymatic contribution of EZH2, like its role in the stabilization of the PRC2 complex [130].

Finally, also Constellation Pharmaceuticals reported a series of benzamide inhibitors that are SAM-competitive. The most active compound of the series inhibited EZH2 with an IC_50_ of 32 nM [131]. In March 2015, they have begun a phase I clinical trial of CPI-1205, a novel inhibitor of EZH2, in patients with B cell lymphomas (ClinicalTrials.gov identifier: NCT02395601). The chemical structure of this inhibitor as not yet been disclosed, it is expected to belong to the pyridone family, similar to the inhibitor CPI-169 (Fig. 3), published by the same research group [118].

## Lysine demethylases

Up to date, two classes of KDM have been described: the amine-oxidase type lysine-specific demethylases 1 and 2 (LSD1 and 2; also known as KDM1A and B, respectively) and the JumonjiC (JMJC) domain-containing histone demethylases. The latter consist of a group which contains over 30 members and can be divided, based on the JMJC-domain homology, into seven subfamilies (KDM2-8) [21, 132, 133]. These two classes of demethylases possess different catalytic mechanism. The LSD-family members are flavin adenine dinucleotide (FAD)-dependent amine oxidases that generate an imine intermediate that is hydrolysed to the demethylated lysine and formaldehyde. Upon recycling of the cofactor FAD, hydrogen peroxide is formed as a byproduct of demethylation. As these enzymes require a free electron pair on the lysine *ε*-nitrogen atom to initiate demethylation, LSD1 and 2 are able to demethylate only mono- and dimethylated but not trimethylated lysines [21]. The Jumonjii domain-containing demethylases are iron and α-ketoglutarate (2-oxoglutarate (2-OG))-dependent enzymes. They are able to remove methyl groups from all three methyl lysine states, with concomitant production of succinate, carbon dioxide, and the demethylated lysine and formaldehyde [134, 135]. The target specificity of KDMs is regulated by their participation in different complexes. KDMs are implicated in different diseases, such as leukemia, prostate and breast cancer, esophageal squamous carcinoma, and as mental retardation [26, 136, 137].

## LSD1/KDM1A

LSD1 bears an amine oxidase-like domain (AOL) at the C-terminal end which displays two folded subdomains: the FAD- and the substrate-binding region. While the FAD-binding subdomain shares many similarities with other FAD-dependent amine oxidases, the substrate-binding subdomain is much larger than in other amine oxidases and is able to accommodate several residues near the target lysine [138]. At the *N*-terminal, the SWIRM domain is important for the protein stability and for the interactions with histone-tails. A tower domain is located within the catalytic center, and it seems to be important for the interaction with other proteins to form complexes, like the co-repressor of RE1-silencing transcription factor (CoREST), HDAC1/2, or the C-terminal-binding protein 1 (CtBP1) [135, 138, 139]. The substrate specificity of LSD1 is influenced by its association with different partners. For example, LSD1 generally demethylates H3K4me1/2, thus repressing gene transcription, but when LSD1 interacts with the androgen receptor (AR), its enzymatic specificity switches to H3K9me1/2, then stimulating transcription [140]. In addition to H3K4me1/2 and H3K9me1/2, LSD1 is also able to demethylate lysines in non-histone proteins like K370 in the transcription factor p53, K185 of E2F1, and K1096 in DNMT1 [141–143]. LSD1 itself in turn is also a substrate for methylation. Dimethylation of LSD1 at lysine (K) 114 by the histone methyltransferase G9A results in the recruitment of the chromatin remodeler chromodomain-helicase-DNA-binding protein 1 (CHD1), which is a key event controlling androgen-dependent target gene transcription and signaling dependent on the TMPRSS2-ERG fusion [144]. Importantly, preventing LSD1 methylation or interaction of CHD1 with methylated LSD1 severely impaired chromatin recruitment of CHD1 and AR, androgen-dependent target gene transcription, chromatin loop formation at the TMPRSS2 locus, and TMPRSS2-ERG gene fusion. This makes targeting of this methylation namely the interaction a promising target for the treatment of prostate cancer.

**Fig. 4:**
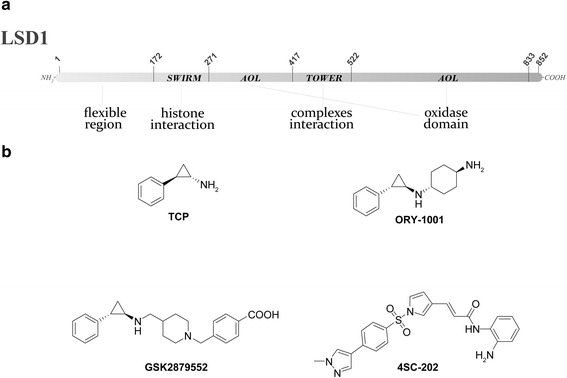
**a** Schematic view of LSD1 principal domains. **b** Structures of the unselective LSD1/MAO inhibitor tranylcypromine (TCP), the selective LSD1 inhibitors from Oryzon and GSK and the dual HDAC/LSD1 inhibitor 4SC-202

As the LSD enzymes are structurally related to the monoaminoxidases MAO-A and MAO-B, some MAO inhibitors, as tranylcypromine (TCP; Fig. 4), an approved drug for the treatment of depression, were among the first discovered KDM1 inhibitors. The TCP is a mechanism-based irreversible inhibitor which bonds to the cofactor FAD [145]. However, the use of unselective compounds as KDM1 inhibitors is limited by their anti-MAO activities. The most common side effects caused by MAOIs include orthostatic hypotension, dizziness, and drowsiness [146, 147]. Moreover, in 1963, Blackwell reported the possibility of hypertensive crises associated with assumption of MAOIs and tyramine-containing foods (like cheeses) [148]. Thus, patients in dose-escalation trials with TCP must be instructed to avoid critical food and have to be monitored intensively to prevent undesired cardiovascular events. TCP was recently reported to inhibit the colony-forming ability of AML cells in a mouse model of MLL-AF9-induced leukemia [149]. It is to note that, in this study, a drug-induced anemia in mice was also reported. Many TCP derivatives have been prepared in order to get more selective LSD1, MAO-inactive compounds [150, 151]. Those would not have the CNS effects of unselective inhibitors and not pose the risk of dangerous interactions with tyramine from food.

Acute promyelocytic leukemia (APL) is a very curable subtype of AML, since APL cells are highly sensitive to all-trans retinoic acid (ATRA). Over 80 % of APL patients can be treated successfully with ATRA-based therapies. For patients with non-APL AML, ATRA has little effect. Consequently, 85 % of these patients will succumb to their disease despite conventional approaches. Little is known about mechanisms of resistance to ATRA in non-APL AML, but data strongly suggest that LSD1 may contribute to ATRA resistance. TCP, as an LSD1 inhibitor, can re-sensitize non-APL AML cells to ATRA [152]. A phase I/II trial of Tretinoin (ATRA, the carboxylic acid form of vitamin A) and TCP was started in September 2014 by a research group of the Martin-Luther-University of Halle-Wittenberg, Germany, in patients with AML who cannot tolerate an intensive chemotherapy (ClinicalTrials.gov identifier: NCT02261779 and EudraCT Number: 2012-002154-23). In October 2014, the University of Miami started a phase 1 study on the safety and tolerability of TCP/ATRA combination therapy in patients with AML and myelodysplastic syndromes (MDS) (ClinicalTrials.gov identifier: NCT02273102). In the trial, increasing doses of TCP (10, 20, 40, and 60 mg) are administrated orally twice a day together with 45 mg/sqm of Tretinoin. In the study of Halle University, patients are treated with daily increasing doses of TCP (initially 10 mg/day, then +10 mg each day up to 80 mg/day) and after 7 days, ATRA is added at a fixed dose (45 mg/sqm/day). The combination of TCP, ATRA, and the chemotherapy agent cytarabine is in a phase I/II study by the University of Freiburg for the treatment of patients with AML and MDS (German Clinical Trials Register, DRKS-ID: DRKS00006055). In the trial, four dose levels of TCP (20, 40, 60, and 80 mg on days 1–28) are examined in combination with fixed dose of ATRA (45 mg/m2 on days 10–28) and fixed dose of cytarabine (40 mg on days 1–10) for the first cycle, for the following cycles, ATRA is administered continuously, except for a 9-day interruption at the beginning of every fourth cycle.

Many TCP derivatives have been reported, some of them, with potency in the low nanomolar range and a very high selectivity over MAOs, were able to induce differentiation in a mouse model of human MLL-AF9 leukemia [149]. Oryzon reported ORY-1001 (Fig. 4), a potent and selective LSD1 inhibitor (IC_50_ of 18 nM and selectivity over MAOs and LSD2 over 1000-fold), which is able to show a time- and dose-dependent H3K4me2 accumulation at KDM1A target genes and induction of differentiation markers in THP-1 cells with MLL translocation (MLL-AF9). It also possesses good oral bioavailability, and daily oral administration of doses lower than 0.020 mg/kg leads to significantly reduced tumor growth in rodent MV(4;11) xenografts [153, 154]. ORY-1001 is currently in a phase I/IIA clinical trial in patients with relapsed or refractory acute leukemia (EudraCT Number: 2013-002447-29). In April 2014, Roche and Oryzon Genomics started a collaboration on LSD1-inhibitors research and Roche will have sole responsibility for developing and commercializing ORY-1001. GlaxoSmithKline reported also a selective LSD1 inhibitor, GSK2879552 (Fig. 4), which entered a phase I study in AML (ClinicalTrials.gov identifier: NCT02177812) and in small cell lung cancer (SCLC) (ClinicalTrials.gov identifier: NCT02034123). GSK2879552 promotes differentiation in AML cells and treatment with this inhibitor resulted in a potent anti-proliferative growth effect in SCLC cells and AML cells. Furthermore, mouse models of AML and SCLC treated with GSK2879552 showed prolonged survival [155]. GlaxoSmithKline has also disclosed a reversible KDM1A inhibitor (GSK354 or GSK690) with both high potency (IC_50_ <100 nM), highly selectivity (MAO IC_50_ >200 μM) and good cellular activity [156]. Additional pre-clinical studies are warranted to validate this compound as a therapeutically promising KDM1A inhibitor.

Interesting is also the use of dual HDAC-LSD1 inhibitors. An example is 4SC-202 (Fig. 4), which inhibits HDAC1/2/3 and LSD1 with similar low micromolar potency. 4SC-202 provokes the inhibition of stemness-related properties of cancer cells and affects their viability [157]. It has, in March 2015, ended a phase I trial in patients with advanced hematological malignancies, and it showed to be well tolerated and to possess anti-cancer activity (ClinicalTrials.gov identifier: NCT01344707) [158]. Very interesting is also the reported synergistic lethal effect against cultured and primary AML blasts showed by the combination of SP2509, a very potent LSD1 inhibitor with panobinostat, a pan-HDAC inhibitor. Compared with each agent alone, co-treatment significantly improved the survival of the mice engrafted with the human AML cells, without exhibiting any toxicity [159].

In December 2015, the Californian company, Imago Biosciences, has announced, for the next year, the beginning of a clinical trial for an oral Imago LSD1 inhibitor for the treatment of myelofibrosis (www.imagobio.com).

## JMJC demethylases

While for LSD1, already four compounds are in clinical trials, the development of clinical candidates against the JMJC domain-containing demethylases is not as advanced. The development of potent and selective JMJC domain-containing demethylases inhibitors is much more complicated. The big challenges come from the high structural similarity of its members and also from the generally poor cellular permeability of the inhibitors since now disclosed (many of which are metal chelators, 2-OG analogs). The KDM5 subfamily, also known as JARID1, demethylates H3K4me2/3; the activities of these enzymes are related with cancer proliferation, reduction of tumor suppressor expression, and drug resistance and relapse [160].

The Danish company, EpiTherapeutics, reported EPT-103182, a small molecule, targeting KDM5B with subnanomolar potency in vitro and a cellular IC_50_ of 1.8 nM in U2OS cells, with 20–50-fold selectivity against KDM4 and 3000-fold against KDM6 [153]. EPT-103182, which structure has not yet been disclosed, is the most advanced KDM inhibitor in preclinical development, it shows an antiproliferative effect in hematological and solid cancer cell lines, and demonstrates dose-dependent tumor growth inhibition in xenograft models [161]. In May 2015, Gilead Sciences has acquired EpiTherapeutics.

Quanticel Pharmaceuticals patented a series of pyridine derivatives as JARID1A (KDM5A), JARID1B (KDM5B), JMJD2C (KDM4C), and FXBL10 (KDM2B) inhibitors (WO 2014100463 A1 and WO 2014151945 A1). The company was recently acquired by Celgene Corporation and first drug candidates from Quanticel are expected to enter the clinic trials in early 2016 (www.quanticel.com).

## Conclusions

Epigenetics provides promising new targets for anticancer therapy. DNA methylation and histone acetylation were already addressed for drug design and several DNA methyltransferases and histone deacetylases inhibitors are FDA-approved anti-cancer drugs. More recently, compounds targeting histone methylation have entered in clinical trials for cancer treatment. In this review, we summarized the last reports in clinical trials for DOT1L, EZH2, and LSD1 inhibitors. EPZ-5676 (pinometostat), a DOT1L inhibitor, is currently in phase I trial in patients with AML with MLL translocation. Even if EPZ-5676 has a low oral bioavailability and the treatment needs to use high drug concentrations, this inhibitor showed promising results in patients afflicted by MLL-rearranged leukemia. EZH2 inhibitors seem to be particularly effective against B cell lymphomas bearing EZH2-activating mutations. GSK126 from GlaxoSmithKline, tazemetostat from Epizyme, and CPI-1205 from Constellation Pharmaceutical are currently in phase I clinical trials for the treatment of this form of NHL. The LSD1 inhibitor TCP could re-sensitize AML cells to ATRA and the Universities of Halle, Miami, and Freiburg are testing the TCP/ATRA combination in patients with AML. Regarding other LSD1 inhibitors, ORY-1001 from Oryzon is in phase I/IIA trial for the treatment of acute leukemia, GSK2879552 is under a phase I clinical trial in patients with AML and SCLC, and 4SC-202, a HDAC1-3 and LSD1 inhibitor ended a phase I trial for hematological malignancies.

This field has just begun to be addressed and, for the moment, the number and the chemical diversity of KMT inhibitors available are limited and, more important, for some KMTs, which could be important targets in cancer therapies (like WHSC1 and KMT2), there are no inhibitors reported yet. Regarding the KDMs, due to the high similar structures of the Jumonji demethylases and the analogy of KDM1 with MAOs, the major challenge is the identification of subtype-selective inhibitors.

Because cross talk can occur between histone methylation and acetylation, a combination of epi-inhibitors targeting these two modifications could represent an interesting approach for future therapeutic intervention. In the last decade, combinations of drugs that modify chromatin or DNA methylation status have already been shown to produce a synergistic reactivation of tumor-suppressor genes and an enhanced anti-cancer effect in several malignancies, like colon [162], cervical [163], and endometrial cancer [164]. Combination therapies are expected to improve the efficacy of the single drugs, in part by limiting acquired resistances and by reducing the side effects through the use of lower dosages of one or both drugs [165]. A combination of the HDAC inhibitor Vorinostat with the LSD1 inhibitor pargyline has recently shown a promising antineoplastic efficacy results in human breast cancer cells [166, 167].

Moreover, the modulation of an aberrant histone methylome profiles could be addressed also through an action on the readers of this modification. This strategy was successful for histone acetylation; in fact, inhibitors of bromodomains, proteins that bind and recognize histone acetylation, are in advanced pre-clinical and clinical studies for the treatment of hematological malignancies [168]. At present, few inhibitors of the histone methylation readers have been reported, but for many targets, no small-molecule ligands are known yet [169]. Recently, we reported the first nanomolar inhibitor of a Tudor domain-containing methyl-lysine reader protein, Spindlin1, which has been reported to be involved in liposarcoma proliferation [170, 171].

Initial results of current clinical trials with drugs targeting the histone methylome will probably guide the future clinical development for new histone methylation modifiers and different therapeutic indications. Still, there is a plethora of targets around histone methylation and demethylation that has not been properly addressed by inhibitors so far, and thus, there will be many further opportunities for epigenetic therapy.

## Abbreviations

2-OG: 2-oxoglutarate, α-ketoglutarate
ALL: acute lymphoblastic leukemias
AML: acute myeloid leukemia
APL: acute promyelocytic leukemia
ATRA: all-trans-retinoic acid
CoREST: co-repressor of RE1-silencing transcription factor
DLBCL: diffuse large B cell lymphoma
DNMT: DNA methyltransferase
DOT1L: disruptor of telomeric silencing 1-like
DZNep: 3-Deazaneplanocin A
EZH2: enhancer of zeste homologue 2
FAD: flavin adenine dinucleotide
FDA: Food and Drug Administration
HDAC: histone deacetylase
KDM: lysine demethylase
KMT: lysine methyltransferase
LSD1: lysine-specific demethylase 1
MAO: monoaminoxidase
MDS: myelodysplastic syndromes
MLL: mixed-lineage leukemia
MTD: maximum tolerated dose
ncRNA: non-coding RNA
NHL: non-Hodgkin lymphoma
SAH: *S*-adenosyl-l-homocysteine
SAM: *S*-adenosyl-l-methionine
PAD, PADI: protein-arginine deiminase
PRC: polycomb repressor complex
PRMT: protein arginine methyltransferase
PTM: post-translational modification
RP2D: recommended phase 2 dose
SCLC: small cell lung cancer
TCP: tranylcypromine
